# Anaphylaxis to selective COX-2 inhibitors: an unexpected culprit

**DOI:** 10.1186/2045-7022-5-S3-P111

**Published:** 2015-03-30

**Authors:** Eunice Dias de Castro, Natacha Santos, Fabrícia Carolino, Josefina R Cernadas

**Affiliations:** 1 Centro Hospitalar de S. João EPE, Porto, Portugal

## Background

Hypersensitivity reactions (HR) to non-steroid anti-inflammatory drugs (NSAID) are frequent and usually related to COX-1 inhibition mechanism. Selective COX-2 inhibitors (sCOX-2i) are commonly safe alternatives.

## Aim

To describe 2 clinical cases of anaphylaxis to sCOX-2i.

## Case Report 1

Male, 40 years-old, non-atopic, who was hospitalized due to anaphylactic shock (nausea, vomiting, dyspnea, hypoxia and hypotension) 30 minutes after etoricoxib intake for low-back pain. He was hospitalized 6 months earlier due to acute respiratory insufficiency with mechanical ventilation of unknown etiology that he later related to etoricoxib intake 60 minutes before. He tolerates paracetamol. Previous tolerance to the strong COX-1 inhibitor diclofenac was confirmed by oral provocation test (OPT).

## Case Report 2

Female, 45 years-old, non-atopic, who was hospitalized due to a biphasic reaction with anaphylactic shock (palmar pruritus, tongue edema, dysphonia, nausea, vomiting, diarrhea, hypotension and lipotimia) 30 minutes after celecoxib intake for low-back pain. Three months earlier, she was observed by her family doctor due to hand pruritus and angioedema, and generalized maculopapular exanthema, 3 hours after celecoxib intake. Previous tolerance to paracetamol and diclofenac was confirmed by OPT. Both patients collected blood samples for quantification of the specific IgE to the culprit sCOX-2i, with results pending.

## Discussion

In our patients, the clinical manifestations and rapid onset are suggestive of anaphylaxis to sCOX-2i. Anaphylaxis to sCOX-2i is rare, with few cases reported with celecoxib, but not etoricoxib, to our knowledge. An allergic mechanism was previously proven with positive lymphocyte transformation tests, but specific IgE antibodies were not reported. Previous cases reported tolerance to rofecoxib and to sulfamethoxazole. In our patients, tolerance to other sCOX-2i as well as drugs with a sulfur moiety have not been tested so far, due to the severity of the reactions with the culprit sCOX-2i and proven tolerance to alternative COX-1 inhibitors.

## Consent

Written informed consent was obtained from the patient for publication of this abstract and any accompanying images. A copy of the written consent is available for review by the Editor of this journal.

